# Effect of Bleaching on Resin-Infiltration-Masked Artificial White Spots In Vitro

**DOI:** 10.3390/jfb15050125

**Published:** 2024-05-13

**Authors:** Alan Leon Sinanovic, Philipp Messer-Hannemann, Mariam Samadi, Falk Schwendicke, Susanne Effenberger

**Affiliations:** 1 Clinical Research, DMG Dental-Material Gesellschaft mbH, 22547 Hamburg, Germany; asinanovic@mtcompanies.com (A.L.S.); pmesser-hannemann@mtcompanies.com (P.M.-H.); msamadi@mtcompnies.com (M.S.); 2 Department Biotechnology, University of Applied Sciences Hamburg, 21033 Hamburg, Germany; 3 Department of Conservative Dentistry and Periodontology, Ludwig-Maximilians University Munich, 80336 Munich, Germany; falk.schwendicke@med.uni-muenchen.de

**Keywords:** resin infiltration, bleaching, caries lesion, color properties, surface roughness

## Abstract

Resin infiltration is an effective method to mask vestibular white spots. If needed, external bleaching is usually recommended before infiltration, whilst in clinical practice, this sequence may not always be feasible. This in vitro study evaluated the effect of bleaching after resin infiltration regarding surface roughness and color using bovine incisors. Unlike for the untreated specimens (control, *n* = 25), artificial caries lesions were created within the test group (*n* = 25) using a demineralization solution at 37 °C for five days (pH = 4.95). The lesions were subsequently infiltrated using a resin infiltrant (Icon, DMG, Hamburg, Germany), followed by polishing. Afterwards, all specimens were bleached with a 10% carbamide peroxide gel (Opalescence, Ultradent, South Jordan, UT, USA) for 8 h/day over a ten-day period. Between bleaching treatments, specimens were stored in an opaque container with moistened paper tissues at 37 °C. Surface roughness was measured using a profilometer, and color in the L*a*b* space was assessed spectrophotometrically before and after bleaching. Bleaching increased the L*-values of both infiltrated (mean ± SD; ΔL* = 3.52 ± 1.98) and untreated (control) specimens (ΔL* = 3.53 ± 2.30) without any significant difference between the groups (*p* = 0.983). Bleaching also induced a significant increase in the mean surface roughness of both infiltrated (*p* < 0.001) and untreated (*p* = 0.0134) teeth. In terms of clinical relevance; it can be concluded that bleaching resin-infiltrated enamel is as effective as bleaching sound enamel.

## 1. Introduction

In recent years, resin infiltration has emerged as a promising treatment option for various dental enamel conditions [1,2,3,4,5,6]. This micro-invasive approach involves the use of a low-viscosity resin to penetrate and fill sub-surface enamel defects, especially masking carious white spots, fluorosis, and hypomineralized lesions [1,4,7,8,9]. Due to the refractive index of the infiltrant (1.47), which is more similar to the refractive index of enamel (1.62) than to those of water (1.33) and air (1.0), which normally fill the microporosities, resin infiltration can enhance the overall esthetic outcome of anterior teeth, providing a conservative alternative to traditional restorative methods [1,3,4,9].

However, one limitation of resin infiltration is its inability to whiten the tooth surface. Studies have suggested that utilizing the synergistic effects of resin infiltration and bleaching can optimize treatment protocols for enhanced esthetic outcomes, ensuring the longevity and esthetic success of the combined approach for anterior teeth [7,10]. Moreover, the interaction between the bleaching agents and the resin-infiltrated lesions themselves is an area of interest. Research focusing on the specific impact of bleaching on resin infiltration, including the potential changes to the microstructure and mechanical properties, is therefore crucial to address these knowledge gaps.

Many studies have shown that bleaching can effectively whiten teeth by removing intrinsic and extrinsic stains [11,12]. In addition to its whitening effects, bleaching has been reported to have other impacts on different composite resins such as restoration materials [13]. For example, one study demonstrated that in-office bleaching can reduce the microhardness of nano-filled composites, while the surface roughness was not significantly altered [14]. Another study showed that in-office bleaching impairs the adhesive strength of composite orthodontic brackets due to oxygen free radicals formed during the treatment [15]. Although the potential effects of bleaching on composite resins were shown in several studies, their clinical relevance remained unclear [13]. Polydorou et al. compared the amount of monomer released from untreated and bleached composite resin specimens. It was shown that a lower amount of Bis-GMA (bisphenol A-glycidyl dimethacrylate) and less UDMA-2 (urethane dimethacrylate) were released from bleached composite resin restorations in comparison to unbleached control samples. In regard to the released concentration of TEGDMA (triethylene glycoldimethacrylate) molecules, there was no difference between the groups [16]. In light of the complexity surrounding the combined treatment of resin infiltration and bleaching, it is also imperative for clinicians to carefully consider the selection of appropriate bleaching agents and techniques, as well as the individual patient factors, before proceeding with such treatment.

Since resin infiltration enables the tooth to recover the natural shade of enamel surrounding the lesion, patients may desire a subsequent bleaching procedure to whiten their teeth. However, it was shown that infiltrated lesions are more susceptible to color alterations compared to different resin bonding agents [17], which raises the question of whether infiltrated lesions can be effectively bleached or not. Furthermore, an increased surface porosity after bleaching of different composite materials was observed, although this effect could only be observed with highly concentrated bleaching agents [18]. Accordingly, there is still a lack of evidence on the impact of low-concentration at-home bleaching agents on infiltrated, non-cavitated lesions.

### Objective

The aim of this study was to evaluate the effects of bleaching resin-infiltrated white spots compared to sound enamel. We aimed to assess the color differences induced by bleaching in infiltrated and non-infiltrated enamel, as well as surface roughness in vitro. We hypothesized that bleaching resin-infiltrated demineralized lesions is as effective as bleaching sound enamel and that there is no difference in the visual appearance between these groups. The null hypothesis was that bleaching resin-infiltrated lesions does not lead to significant color alterations.

## 2. Materials and Methods

### 2.1. Experimental Setup

**Figure 1:**
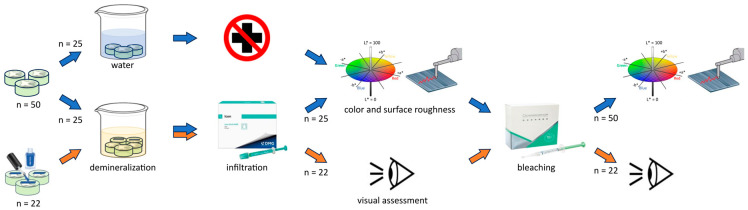
Depiction of the experimental setup. The blue arrows show the sequence of the instrumental measurement, and the orange arrows show the sequence of the visual assessment. Figure modified from Jacob et al. [19].

The description of the experimental setup is shown in Figure 1. In short, bovine teeth were embedded, ground, polished and subsequently divided into three groups: control (*n* = 25), test (*n* = 25), and visual assessment (*n* = 22). Specimens in the control group were left untreated (stored in water at 10 °C), whereas in the test group, artificial lesions were created and subsequently infiltrated. The color and surface roughness were measured before and after bleaching of the specimens in both groups. Half of the exposed surface of the specimens belonging to the visual assessment group was covered with blue nail polish before the artificial lesions were created and subsequently infiltrated to visually compare a bleached infiltrated enamel surface and a bleached untreated enamel surface. For this purpose, the nail polish was removed by mechanical polishing before the bleaching treatment. A visual inspection was carried out to ensure that the nail polish had been completely removed before bleaching. The experimental procedures are further outlined in the following chapters.

### 2.2. Specimen Preparation

Bovine incisors (*n* = 72) were acquired already prepared (cleaned, root cut off, and pulp removed) from a local supplier of tooth samples (Rocholl GmbH, Eschelbronn, Germany; officially registered with the veterinary authorities for the preparation of bovine teeth). Cylindrical molds were used, which were sprayed with silicone (Weicon, Münster, Germany). Teeth were placed on their vestibular side in the middle of the mold and embedded in a 2-component cold-polymerizing resin (Technovit 4004, Kulzer, Wehrheim, Germany). For this, the liquid and powder of the embedding resin were mixed in a 1:1 ratio and poured into the cylindrical molds. After the resin had hardened, specimens were ground and polished.

### 2.3. Grinding and Polishing of the Specimens

For grinding the specimens, a grinding/polishing machine (TegraPol-21, Struers, Willich, Germany) with a sandpaper disk (SiC Paper #320, Struers, Willich, Germany) was used. A speed of 150 rpm and synchronous direction was set, with a force of 15 N, a duration of 10 s, and water flow, to expose a tooth enamel area of approx. 64 mm^2^.

With the grinding/polishing machine, the specimens were polished using the scheme in Table 1. After polishing, the prepared specimens were stored at 10 °C in water.

### 2.4. Color Measurement

The color parameters of the specimens were determined using a spectrophotometer (CM-36dGV, Konica Minolta, Tokyo, Japan) before and after bleaching. This was done via a reflection measurement in which the intensity of the reflected light was measured. The data were converted into the quantitative color specification system L*a*b*. The mean values and the color difference Δ*E* before and after bleaching were calculated using the following formula.

(1) $$∆E=\sqrt{{\left(L_{t_{2}}^{*}-L_{t_{1}}^{*}\right)}^{2}+{\left(a_{t_{2}}^{*}-a_{t_{1}}^{*}\right)}^{2}+{\left(b_{t_{2}}^{*}-b_{t_{1}}^{*}\right)}^{2}}$$

### 2.5. Surface Roughness Measurement

A profilometer (Marsurf PS1, Mahr, Göttingen, Germany) was used to determine and evaluate the surface roughness of the specimens. The surface was scanned six times over a measuring length of 1.75 mm in the form of triangles to determine a larger area of the surface. The enamel surface is an aperiodic profile, which is why the arithmetic mean roughness value *R*_A_ (≤0.1 µm) and the mean roughness depth *R*_Z_ (≤0.5 µm) were considered for the assessment. This corresponds to an evaluation length of 1.25 mm and a sampling length of 0.25 mm.

### 2.6. Creation of Artificial Lesions

The creation of artificial lesions was performed based on the studies of Buskes et al. on lesion formation by demineralization [20]. All solutions were prepared in distilled water, and the compositions are shown in Table 2.

A volume of 120 mL of demineralization solution per 64 mm^2^ of exposed enamel surface was determined. Test specimens were placed in opaque containers with the corresponding volume of demineralization solution. The pH value of the solution was set to 4.95, and the specimens were incubated at 37 °C for 5 days. The pH value was measured daily and adjusted with hydrochloric acid or potassium hydroxide if necessary.

### 2.7. Resin Infiltration Treatment

Artificial lesions were infiltrated with a low-viscosity resin (Icon, DMG, Hamburg, Germany) using a modified protocol compared to the manufacturer’s instructions. While the manufacturer recommends 2 min of etching with 15% HCl (Icon-Etch, DMG, Hamburg, Germany) to remove the pseudo-intact surface in a clinical setting, etching with 37% phosphoric acid is recommended for artificial lesions in bovine teeth as no hypermineralized surface is present that must be removed [21]. The surfaces of the specimens were thus etched with 37% phosphoric acid (Etching Gel, DMG, Hamburg, Germany) for 5 s. Following the removal of the etching gel, the surface was rinsed with water for 30 s. Next, the surface was dried using Icon Dry (DMG, Hamburg, Germany) for 30 s before applying the resin infiltrant for 3 min. After light curing, the resin infiltrant was applied a second time for 1 min and subsequently light cured. Specimens were polished with polishing discs (Flexipol-Set, NTI-Kahla, Kahla, Germany) using the following parameters: a bur speed of 12,000 rpm, a motor speed of 2400 rpm, and a gear ratio of 1:5, aiming to show an optical gloss on the enamel surface in the operating light.

### 2.8. Bleaching Treatment

The specimens of the control and test group were whitened with a 10% carbamide peroxide gel (Opalescence, Ultradent, Cologne, Germany), which was applied with an application brush to the exposed enamel surface. The specimens were then stored in an opaque container with moistened cloths at 37 °C for eight hours. After the incubation period, the containers were removed from the cabinet and the specimens were cleaned with a toothbrush and water. The cleaned specimens were then stored in the containers with moistened cloths at 37 °C for 16 h until the next bleaching treatment. The bleaching treatment was repeated for ten days according to the manufacturer’s instructions.

### 2.9. Visual Assessment

In order to directly compare the bleached infiltrated and untreated enamel surfaces, a visual assessment was carried out by one experienced operator according to Figure 1. This was done by a visual assessment of the difference between the treated surface areas using an analog scale ranging from 1 to 7: no difference (1, 2); little difference (3); medium difference (4, 5); noticeable difference (6, 7). Bleaching was then carried out, and the specimens were evaluated again.

### 2.10. Statistical Analysis

We tested for a normal distribution using the Kolmogorov–Smirnov test and applied pairwise and independent two-sided t-tests for comparing groups (Excel 2021, Microsoft Corporation, Redmond, WA, USA). The significance level was set at 5% (*p* = 0.05).

## 3. Results

### 3.1. Evaluation of the Color Measurement

The luminance L* in the CIE-Lab system represents the brightness value of a color and indicates the dimension from darkness (0) to brightness (100). As bleaching results in tooth brightening, ΔL* and Δ*E* are primarily considered when analyzing the shade differences. Table 3 shows that there was a significant brightening (difference in L*) of the control teeth after the bleaching treatment (*p* < 0.001). In addition, the mean color difference Δ*E* between before and after bleaching exceeded the clinical acceptable thresholds found in the literature for color differences perceptible to the human eye [22,23].

Bleaching of the test specimens significantly increased the luminance (L*) values of the test group (*p* < 0.001) and thus resulted in successful whitening. The color difference Δ*E* between before and after bleaching also exceeded the clinical acceptable threshold. The changes in brightness (ΔL*) for the control and test groups did not significantly differ from each other after bleaching (*p* = 0.983); thus, comparable whitening was achieved. However, the data show that the color difference for the untreated specimens after bleaching was greater than that for the resin-infiltrated specimens, but again, this difference was not significant (*p* = 0.679).

### 3.2. Evaluation of the Surface Roughness Measurement

Table 4 shows the arithmetic mean roughness value *R*_A_ and the average roughness depth *R*_Z_ of the control and test group before and after bleaching.

Bleaching significantly increased the *R_Z_* value of the control group (*p* = 0.0134). The *R_A_* value also increased after bleaching, but not statistically significantly (*p* = 0.0975). Bleaching of the resin-infiltrated specimens in the test group led to a significant increase in both the arithmetic mean roughness *R*_A_ and the average roughness depth *R*_Z_ (*p* < 0.001).

The specimens in the test group showed a higher surface roughness after infiltration compared to the untreated specimens in the control (*p* < 0.001). A significant difference between the groups was also observed after bleaching (*p* < 0.001).

### 3.3. Evaluation of the Visual Assessment

**Figure 2:**
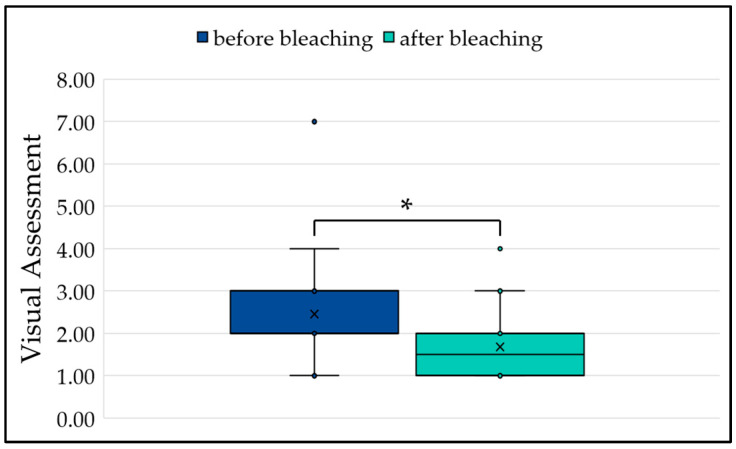
Comparison of the differences between the areas before and after bleaching. ‘x’ denotes the respective mean value and ‘*’ the significance at *p* < 0.05.

Based on the visual assessment of the specimens before and after bleaching, the results show that infiltration of the uncovered side led to a small but still visible difference compared to the untreated side of the specimen. After bleaching, no visible difference was seen between the untreated and infiltrated areas, meaning that a significant change (*p* = 0.0239) was achieved through bleaching of the infiltrated side of the specimen (Figure 2).

## 4. Discussion

Based on the results and considering the limitations, such as the use of bovine teeth, the treatment of artificial lesions, and the use of phosphoric acid instead of hydrochloric acid etching gel, bleaching after a resin infiltration treatment proved to be promising. The infiltrated and untreated specimens achieved comparable whitening after bleaching. By infiltrating the microporosities of the demineralized enamel, the lesions were penetrated with a low-viscosity resin. Although resin-based materials are widely used in restorative dentistry, they are subject to color change over time [24,25]. In particular, TEGDMA, the main component of the infiltrant used in this study, tends to increase water absorption and potential changes in color over time [26,27]. However, recent studies showed that infiltrated and stained enamel lesions can be satisfactorily bleached, indicating that the visual appearance can be recovered [27,28,29].

Furthermore, the present study found that the color of the infiltrated surface matched that of the untreated enamel surface following the bleaching treatment, resulting in no significant difference being visible. These results are in accordance with those from Jacob et al. [19]. Since a numerically small Δ*E* does not necessarily correspond to visual perception, as the eye does not react uniformly to differences in hue, color value, and chroma [30], an additional visual assessment was carried out in this study, which was able to confirm the results of the spectrophotometric measurements. Bleaching also allows the color of the infiltrant to adapt to the color of the enamel for a better esthetic appearance. These findings are in accordance with those from Rocha et al., who illustrated a positive bleaching effect on infiltrated teeth [31]. As the masking effect after resin infiltration of non-cavitated lesions is well known and the aim of this study was to evaluate only the effect of bleaching infiltrated lesions compared to sound enamel, it was decided that data on the untreated condition in the test group were not necessary for evaluating the hypothesis of this study. While an increase in the L* value was observed after bleaching, indicating greater brightness, the a* and b* values of the untreated and infiltrated samples tended to decrease, indicating lower color intensity. These results are consistent with those from previous studies in which similar color changes were observed in bovine and human teeth after bleaching treatments [32,33,34]. A regression of the a* and b* values towards zero indicates a change towards an achromatic shade, which improves the visual perception of the tooth to a “whiter” shade after bleaching.

Regarding the surface roughness of the groups, it was shown that resin infiltration increases both the average roughness depth and the arithmetic mean roughness value when compared to an untreated polished enamel surface. However, this increase might also be attributed to the demineralization process that is assumed to alter the surface structure of the specimen. In contrast to the untreated samples, which were subjected to machine polishing, the infiltrated samples were manually polished to better mimic the clinical situation. This may have also affected the surface roughness of the specimens. Bleaching led to a further increase in surface roughness. This effect was also observed by Jacob et al., where bleaching led to moderate and irregular morphological changes [19]. The findings of this study also align with those of a split-tooth, in vitro study that used bovine incisors and a similar study design [33]. Their results showed no clinically significant difference in ΔE between bleached resin-infiltrated teeth and bleached sound enamel. Additionally, the study found an increase in surface roughness when bleaching followed resin infiltration, which is consistent with the results of this study. However, it is important to note that an in-office bleaching system with 40% hydrogen peroxide was used instead of the 10% carbamide peroxide gel that was used in this study. Since bovine enamel was used instead of human enamel in this study, the etching protocol had to be changed. An etching procedure with phosphoric acid for 5 s was chosen, as this protocol had already been tested in previous studies with bovine teeth [8,21,28]. Previous studies stated that inconsistent data exist regarding whether bovine teeth can be considered an appropriate substitute for human teeth [35]. However, it was found that bovine and human enamel substrates behave similarly in terms of staining and bleaching effects [36].

There are currently not enough studies that have investigated the surface properties of bleached resin-infiltrated teeth. However, studies that have examined the surfaces of resin composites show that roughening can be expected after bleaching [37,38]. Further investigations will examine the effects of bleaching-induced surface roughening on the accumulation of biofilm or additional deposits.

## 5. Conclusions

The results of this study suggest that bleaching resin-infiltrated enamel with a low-concentration bleaching agent is just as effective as bleaching sound enamel. It can therefore be concluded that resin infiltration is not a limiting factor for patients who desire tooth whitening. However, bleaching also led to a significant increase in the average surface roughness of both infiltrated and untreated samples. The clinical relevance of this will be investigated in future studies based on biofilm accumulation.

## Figures and Tables

**Table 1:** Polishing settings.

Parameter	Step 1	Step 2	Step 3
Polishing disk	MD-Largo	MD-Floc	MD-Nap
Dosing unit	1/Niveau 8	0/Niveau 10	-
Diamond suspension	9 μm	3 μm	Detergent
Pressure	7–8 bar	7–8 bar	7–8 bar
Speed	150 rpm	150 rpm	150 rpm
Direction	Counterwise	Synchronous	Counterwise
Force	15 N	15 N	15 N
Duration	180 s	120 s	45 s
Water	Off	Off	On

**Table 2:** Composition of the demineralization solution.

Name	Substance	Concentration
MHDP Stock Solution	Methylenediphosphonic Acid	0.5 g/L
KOH Stock Solution	Potassium Hydroxide Pellets	485.9 g/L
Demineralization Solution	Calcium Chloride Potassium Dihydrogen Phosphate MHDP Stock Solution Acetic Acid 99% KOH Stock Solution Thymol	0.3 g/L 0.4 g/L 2 mL/L 3 mL/L 5 mL/L 0.6 g/L

**Table 3:** Color values of the specimens before and after bleaching.

Group		Before Bleaching			After Bleaching				
		L*	a*	b*	L*	a*	b*	ΔL*	Δ*E*
**Control**	Mean	81.80	0.39	14.88	85.35	−0.96	10.88	3.53	6.08
	SD	2.75	1.35	2.18	1.71	0.86	1.64	2.30	2.05
**Test**	Mean	81.57	3.35	17.07	85.09	2.02	14.63	3.52	4.83
	SD	2.78	1.07	2.52	2.41	0.89	2.01	1.98	2.29

**Table 4:** Mean roughness *R*_A_ and roughness depth *R*_Z_ of the specimens before and after bleaching.

Group		Before Bleaching		After Bleaching	
		*R*_A_ [μm]	*R*_Z_ [μm]	*R*_A_ [μm]	*R*_Z_ [μm]
**Control**	Mean	0.032	0.28	0.043	0.39
	SD	0.026	0.16	0.019	0.12
**Test**	Mean	0.056	0.43	0.079	0.62
	SD	0.011	0.08	0.028	0.18

## Data Availability

The data that support the findings of this study are available from the corresponding author upon reasonable request.
